# CASE REPORT Management of Periauricular and Auricular Necrotizing Wound From Brown Recluse Spider Bite Using Negative Pressure Wound Therapy and Wound Interface Modulation

**Published:** 2012-06-07

**Authors:** Mark Chariker, Rachel Ford, Erik Rasmussen, Elaine Schotter

**Affiliations:** Division of Plastic and Reconstructive Surgery, Department of Surgery, University of Louisville, Louisville

Brown recluse spider (*Loxosceles reclusa*) envenomation, known as loxoscelism, can produce a wide range of consequences. The symptoms may range from itching to wound necrosis, which may ultimately result in death.^1^ Biopsies of loxoscelism wounds show evidence of acute inflammation, hemorrhage, thrombosis, induration, and liquefactive necrosis of the dermis and the epidermis.^2^ These effects are attributed to proteolytic toxins in the venom that degrade tissue and cause gravitational spreading of the lesion and dysregulated activation of neutrophils, which is suspected to be involved in dermonecrosis.^2^ Treatment includes early recognition, cool compresses for mild itching, a possible trial of the leukocyte inhibitor dapsone, and hyberbaric oxygen for chronic lesions.^1^ The resultant wound, especially in the head and neck area, necessitates innovation to maintain the tenants of open wound care including moist wound care, prevention of normal adjacent skin maceration, protection of critical structures in the area, and creation of a dynamic wound environment. These tenants are also features of negative pressure wound therapy (NPWT) dressings. We describe the use of this type of dressing for a necrotizing wound caused by a brown recluse spider bite.

There is a paucity of literature that illustrates the use of NPWT specifically on auricular wounds. No articles were discovered during the research for this article that documented the use of NPWT on an ear with an intact external auditory canal.^3^ It is plausible that the application of negative pressure over a wound that includes the auditory canal could potentially perforate the tympanic membrane. Although no perforations have occurred in our institution, the use of negative pressure directly over the ear canal and the tympanic membrane is painful for children and has prevented the use of NPWT in this area. One technique used by clinicians using NPWT is adding a contact layer over certain structures that modulate or, if needed, block the effect of the negative pressure on the surface of delicate structures such as the tympanic membrane or eye. By changing or adding a contact layer, the wound interface strain or deformations on the surface are decreased or blocked. We refer to this NPWT technique as wound interface modulation, and this article describes the use of wound interface modulation to protect the tympanic membrane from the negative pressure applied to the wound in the management of a periauricular and auricular necrotizing wound.

## CLINICAL REPORT

**Figure 1:**
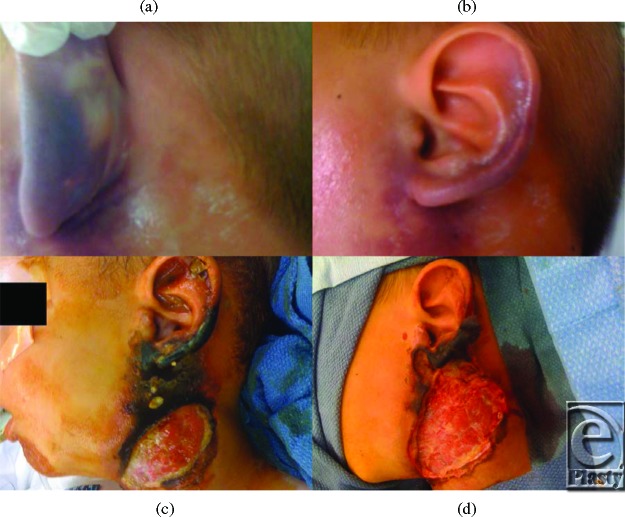
(a and b) Initial presentation of the wound. (c and d) Progression of the wound necrosis.

**Figure 2:**
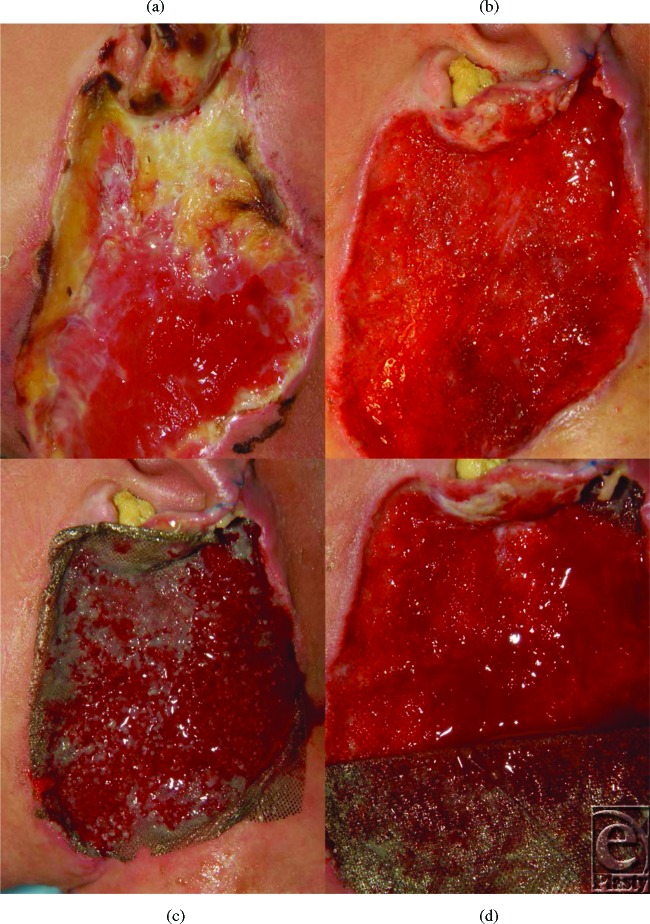
(a and b) The wound after initial debridement. (c and d) A single layer of Acticoat was used as a contact layer for the negative pressure wound therapy dressing.

A 5-year-old white boy presented with soft-tissue cellulitis involving the soft tissue of the lower ear auricle and adjacent neck (Figs 1*a* and 1*b*) as a result of a brown recluse spider bite. Wound cultures gave negative results upon initial debridement. The involved area progressed from cellulitis to necrosis of the skin of the lateral neck and lower auricular cartilage (Figs 1*c* and 1*d*). The initial management consisted of intravenous antibiotics and debridement. The plastic surgery service was consulted on day 12. The wound was debrided again (Figs 2*a* and 2*b*) and cultures returned with methicillin-resistant *Staphylococcus aureus* sensitive to vancomycin and trimethaprim/sulfamethoxazole. Negative pressure wound therapy was started immediately following the debridement on day 12. Nanocrystalline silver mesh (Acticoat; Smith & Nephew, St. Petersburg, FL) without the absorbent layer was used as a contact layer (Figs 2*c* and 2*d*), and polyurethane foam was used as the wound filler.

**Figure 3:**
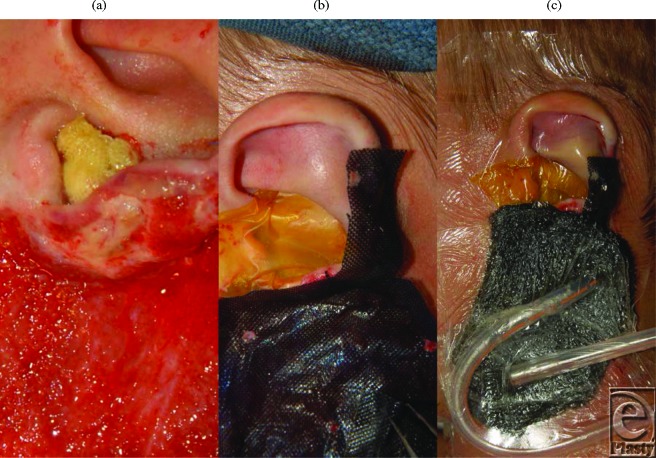
These images demonstrate the obstruction of the ear canal with Xeroform. (a) Gauze covered with an occlusive dressing. (b) The lower part of the wound is covered with a single layer of Acticoat, which serves to modulate the wound as well. (c) The entire dressing effectively controls the wound dynamics in a difficult location. Note the second catheter placed to irrigate the wound if heavy exudate is discovered.

**Figure 4:**
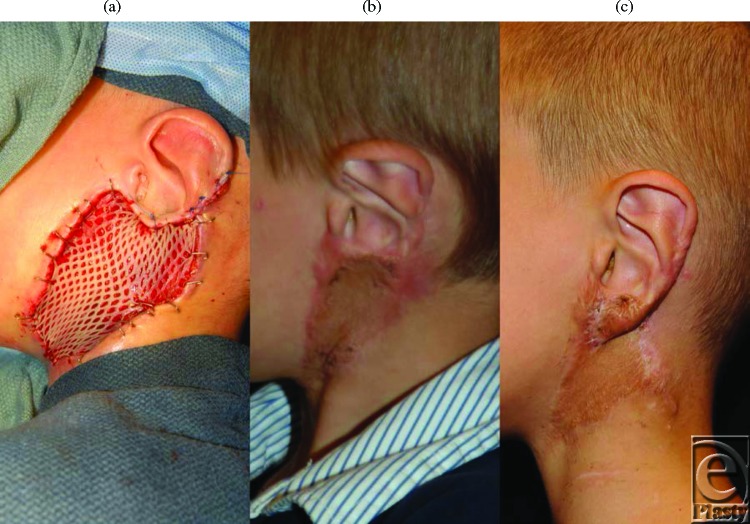
These images show the wound after negative pressure wound therapy treatment and the application of a meshed, split-thickness skin graft. The patient is awaiting ear reconstruction. (a) First stage wound closure with skin graft, (b) 1 year after wound closure, and (c) 3 months after first stage ear sulcus and lobule restoration.

Because of the difficulty in maintaining a vacuum seal, the NPWT dressing was placed over the entire ear auricle. The external auditory canal was dressed with Xeroform gauze (Fig 3*a*), and the auricle was incorporated into the dressing (Fig 3*c*). The dressing was irrigated with sterile water daily through an irrigation tube both to promote exudate clearance and to activate the silver in the dressing. The polyurethane foam was placed directly adjacent to the canal beneath the overlying occlusive dressing (Fig 3*c*). The wound interface pressure was set to -80 mm Hg, and the dressing was changed and the wound debrided at 3- to 5-day intervals in the operating room. The wound bed was fully granulated after 20 days from the initial debridement and required a total of 7 days of NPWT. It was closed with a meshed, split-thickness skin graft (Figs 4*a*–4*b*), and the patient had an uneventful recovery. The patient had a first-stage reconstruction of the ear lobule at 14 months after the injury (Fig 4*c*) and will undergo 1 more procedure to complete the sulcus restoration and removal of more of the split-thickness graft.

## DISCUSSION

Delicate structures such as the tympanic membrane are subject to injury from a negative pressure strain. In the pediatric population, the strain also results in pain and dressing intolerance. This injury and pain can be prevented by the use of an occlusive layer that blocks the transduction of the negative pressure to the structure. In this case, multilayer petrolatum gauze was placed inside the auditory canal and a nonpermeable occlusive dressing placed over the canal. This alteration made it possible to then cover the entire ear with the overlay part of the dressing, taking advantage of the ease of contact with the flat skin around the ear. This wound interface modulation is useful around the eye and the nose as well. Once the wound is granulating, other conventional dressings are optional.

In the open segment of the wound, wound interface modulation was used to modify the wound surface in preparation for grafting. The granulating surface was covered with a sheet of Acticoat without the absorptive layer (Fig 3*b*). This layer consists of polyethylene coated with nanocrystalline silver. Because of the pore size and silver, the wound surface is affected both by lower negative pressure and by topical silver therapy.^4^^,^^5^ The effects on the granulation tissue are not clear at present, but the granulation bed appears less hypertrophic.

This study highlights the protective effects of an occlusive dressing placed in the external auditory canal when negative pressures are being applied to tympanic membranes. Since the amount of negative pressure needed to rupture a human tympanic membrane is not known at this time, we recommend the use of low negative pressures (such as -80 mm Hg used in this case) even with an occlusive dressing. It should be noted that the use of -80 mm Hg pressure in this case report still resulted in a favorable result. The issue of patient comfort is also a factor. This small innovation arose from the discomfort and pain experienced by children when this technique was used. This technique also allows the use of the intermittent mode, which is otherwise intolerable.

The mechanism of action of NPWT can be categorized into wound strain, wound perfusion, wound drainage, and wound maintenance. All components are affected by the wound contact layer, which may override parameters of the wound filler.^6^
